# Artificially Increasing Cortical Tension Improves Mouse Oocytes Development by Attenuating Meiotic Defects During Vitrification

**DOI:** 10.3389/fcell.2022.876259

**Published:** 2022-03-24

**Authors:** Xingzhu Du, Jun Li, Qingrui Zhuan, Luyao Zhang, Lin Meng, Panyu Ren, Xiaohan Huang, Jiachen Bai, Pengcheng Wan, Wenquan Sun, Yunpeng Hou, Shien Zhu, Xiangwei Fu

**Affiliations:** ^1^ Key Laboratory of Animal Genetics, Breeding and Reproduction of the Ministry of Agriculture and Rural Affairs, National Engineering Laboratory for Animal Breeding, Beijing Key Laboratory for Animal Genetic Improvement, College of Animal Science and Technology, China Agricultural University, Beijing, China; ^2^ Department of Reproductive Medicine, Reproductive Medical Center, The First Hospital of Hebei Medical University, Shijiazhuang, China; ^3^ State Key Laboratories of Agrobiotechnology, College of Biological Sciences, China Agricultural University, Beijing, China; ^4^ Institute of Biothermal Science and Technology, School of Medical Instrument and Food Engineering, University of Shanghai for Science and Technology, Shanghai, China; ^5^ State Key Laboratory of Sheep Genetic Improvement and Healthy Breeding, Institute of Animal Husbandry and Veterinary Sciences, Xinjiang Academy of Agricultural and Reclamation Sciences, Shihezi, China

**Keywords:** oocyte vitrificaion, cortical tension, meiosis, spindle assembly checkpoint, aneuploidy

## Abstract

Oocyte cryopreservation demonstrates great benefits in the conservation of animal germplasm resources and assisted reproductive technology. However, vitrification causes damages in oocytes, which would lead to the decrease of oocyte quality, and embryonic development post fertilization. Cytoskeleton plays an important role in regulating cell shape, organelle migration, cell division and mechanical signal transduction. Cortical tension is a reflection of the physiological state and contractile ability of cortical cytoskeleton. Appropriate cortical tension is prerequesite for normal oocyte meiosis. In the present study, oocyte cortical tension was examined by evaluating the levels of cortical tension-related protein pERM (Phospho-Ezrin/Radixin/Moesin) and pMRLC (Phospho-Myosin Light Chain 2). We found that the cortical tension of vitrified oocytes was decreased. Increasing cortical tension of vitrified oocytes by adding 10 μg/ml ConA during *in vitro* culture could significantly improve the polar body extrusion rate and embryo development. Furthermore, increasing the cortical tension could improve spindle positioning, maintain kinetochore-microtubule (KT-MT) attachment, strengthen spindle assembly checkpoint (SAC) activity, and reduce the aneuploidy rate in vitrified oocytes. In conclusion, vitrification induced a remarkable decrease in cortical tension, and increasing the cortical tension could rescue the meiosis defect and improve oocyte quality.

## Introduction

In oocyte meiosis, errors in chromosomes segregation generate eggs with an abnormal number of chromosomes. When fertilized, these eggs lead to aneuploid embryos. It is well known that aneuploidy would induce severe cellular dysfunction since each chromosome encodes thousands of genes (Webster and Schuh, 2017). Oocyte chromosome (or bivalent) segregation errors are usually classified to chromatids premature separation and chromosomal non-disjunction during oogenesis (Mihajlovic and FitzHarris, 2018). Chromosome cohesion defects, kinetochore-microtubule (KT-MT) unattachment and spindle assembly checkpoint (SAC) deficiency are the main factors contributed to chromosome segregation errors (Lane and Jones, 2014; Nakagawa and FitzHarris, 2017; Greaney et al., 2018; Gruhn et al., 2019).

Cellular mechanics emerges as a crucial regulator in cell division, migration and metabolism in recent years (van Helvert et al., 2018; Sitarska and Diz-Munoz, 2020; Romani et al., 2021). Mechanical signals play an important role in the development of ovaries, follicles and oocytes (Shah et al., 2018; Nagamatsu et al., 2019; Pennarossa et al., 2021). Cortex is located under the plasma membrane, and one of its most basic functions is to regulate the shape and mechanical properties of the cell (Taneja et al., 2020). Cortical tension is a highly sensitive readout of contractility in the cortical cytoskeleton and reflects the biochemical and structural features of the cortex, which are mediated by actin assembly, organization of actin polymers, myosin-II motor activity, and linkages between the polymers and the membrane (Evans and Robinson, 2011). Ezrin/Radixin/Moesin (ERM) protein family and non-muscle myosin-II play an important role in regulating cortical tension in oocytes, being involved in cell polarity establishment and promote meiosis progress by regulating the mechanical properties of oocytes (Larson et al., 2010). ERM functions in the active phosphorylated-ERMs (pERM) form and Myosin-II activity is partly regulated by phosphorylation of its regulatory light chain (pMRLC) (Vicente-Manzanares et al., 2009; Fehon et al., 2010).

In mouse oocytes, the cortical tension drops about 6-fold from prophase I to metaphase II and then increases 1.6-fold upon fertilization, indicating that cortical remodeling and the change of mechanical properties during oocyte development are important for meiosis progress (Larson, et al., 2010). During spindle positioning, F-actin forms an actin cage that surrounds the spindle and connects it to the thick actin cortex (Azoury et al., 2008; Schuh and Ellenberg, 2008). Myosin-II pulls the spindle towards the cortex by pulling on filaments emanating from the cortical thickening overlapping with filaments from the actin cage. Therefore, the pulling force depend on the overlap between cortical and cytoplasmic filaments (Verlhac et al., 2000). The fluorescence intensity of F-actin between the leading pole and cortex was higher than that on the lagging pole side. As the spindle moves toward the cortex, the overlap on the leading pole side increases, causing the spindle to move faster. Importantly, there is a narrow window: too low or too high cortical tension both lead to unsuccessful spindle positioning (Chaigne et al., 2015).

Oocyte cryopreservation has attracted worldwide attention in recent years for its significance in fertility preservation (Huang et al., 2019; Bosch et al., 2020; Bojic et al., 2021). Vitrification is an ultrafast cooling method performed with a very high concentration of cryoprotectant for dehydration to avoid the formation of ice crystals (Rall and Fahy, 1985). Vitrification has higher cell survival, fertilization, embryo development and pregnancy rates compared with traditional slow-freezing (Valojerdi and Salehnia, 2005; Saragusty and Arav, 2011; Rienzi et al., 2017). However, studies have revealed that vitrification could cause damage to zona pellucida (Larman et al., 2006), membrane permeability (Zeron et al., 2002; Zhang et al., 2021), cytoskeleton (Schmidt et al., 2004; Tamura et al., 2013; Wei et al., 2013), chromosome (Cheng et al., 2014) and mitochondria (Yan et al., 2010; Manipalviratn et al., 2011; Gao et al., 2019), which eventually impaired subsequent development.

At present, the dynamic alteration of cortical tension in response to vitrification and the effect of cortical tension on oocyte development have not been identified. In the present study, we found that vitrification decreased the oocyte cortical tension. Artificially increasing cortical tension in vitrified oocyte could increase oocyte and embryo development. Furthermore, it could improve spindle positioning, SAC activity, KT-MT attachment, and reduce aneuploidy rate. Whether this mechanism is conserved in non-rodent animals needs further study. Our results will provide an interdisciplinary perspective into understanding the cryoinjuries in mouse oocytes, and provide a reasonable theoretical basis for further research in delineating the mechanism underlying cortical tension-mediated meiosis progression.

## Materials and Methods

### Ethics Statement

All chemicals were purchased from Sigma Chemical Co. (St Louis, MO, United States) unless otherwise stated. All animal manipulations were performed according to the guidelines of the Animal Care and Use Committee. The present study was approved by the Institutional Animal Care and Use Committee of China Agricultural University (AW01040202-1).

### Mouse

Studies were performed using 8-week-old female mouse (CD-1^®^ (ICR)) (Vital River Laboratory Animal Technology Co., Ltd. Beijing, China). Mouse was housed in ventilated cages on a 12 h light/12 h dark cycle (lights on from 08: 00 to 20: 00) under controlled temperature (22 ± 2°C) with food and water freely available. Mouse was allowed to adapt to conditions for 7 days before the initiation of experiments.

### Oocyte Collection

To collect immature oocytes, mouse was sacrificed by cervical dislocation 48 h after they were injected with 10 IU pregnant mare serum gonadotropin (PMSG). Germinal vesicle (GV) stage oocytes were collected by removing cumulus cells in a drop of M2 medium supplemented with dbcAMP (100 ng/ml) through repeatedly pipetting. Then GV oocytes were *in vitro* matured in the M16 medium at 37°C with 5% CO_2_. To collect *in vivo* matured oocytes, mouse was superovulated using10 IU PMSG, followed by injection with 10 IU human chorionic gonadotrophin (hCG) 48 h later to induce superovulation. At 13–14 h post-hCG injection, cumulus-oocyte complexes (COCs) were retrieved from the ampulla. Oocytes were collected in M2 medium, the cumulus cells were removed enzymatically using 0.1% (w/v) hyaluronidase.

### Oocytes Vitrification and Warming

Vitrification and warming procedures were conducted as described previously (Huang et al., 2019). For vitrification, pretreatment solution was PBS medium contained 10% (v/v) dimethylsulfoxide (DMSO) and 10% (v/v) ethylene glycol while vitrification solution (EDFS30) was PBS medium contained 30% Ficoll (w/v), 15% EG (v/v) and 15% DMSO (v/v) in 0.5 M sucrose. GV and MII Oocytes were vitrified in EDFS30 by the open pulled straws (OPS) method. First, oocytes were rinsed in pretreatment solution for 30 s, then transferred to vitrification solution in the narrow end of the OPS, and held for 25 s. Then the straws were immediately plunged into liquid nitrogen (LN_2_). Vitrified oocytes were stored in LN_2_ for at least 1 week. For thawing, the oocytes were rinsed in 0.5 M sucrose for 5 min, then rinsed three times in M2 medium. Vitrified-thawed oocytes were recovered 1 h in M2 medium supplemented with dbcAMP (100 ng/ml) before culturing.

### *In vitro* Maturation of Oocytes

GV oocytes were maturated in M16 medium under mineral oil, maintaining in 5% CO_2_ with maximum humidity at 37°C. 10 μg/ml Concanavalin A (ConA) (Sigma, C7642) was added during the entire IVM (*in vitro* maturation) process. A previous study has shown ConA could penetrate zona pellucida intact oocytes (Chaigne et al., 2013). Polar body extrusion (PBE) rate was recorded at 10 h after GVBD.

### Parthenogenesis Activation of Oocytes

The denuded oocytes were transferred first into (Ca^2+^)-free human tubal fluid (HTF) medium supplemented with 10 mM strontium chloride (SrCl_2_) and 5 μg/ml cytochalasin B, incubated at 37°C with 5% CO_2_ for 2.5 h. Then oocytes were transferred into HTF with 5 μg/ml cytochalasin B, incubated at 37°C with 5% CO_2_ for 3.5 h. Activated oocytes were then cultured in a KSOM medium at 37°C with 5% CO_2_ for early embryo development. Cleavage and blastocyst rates were recorded at 24 and 96 h after activation, respectively.

### Immunofluorescence Staining and Chromosome Spread

Oocytes were fixed with 4% (w/v) paraformaldehyde (PFA) for 40 min at room temperature, followed by permeabilization with 0.5% Triton X-100 at room temperature for 1 h. After being blocked in 3% BSA for 1 h at room temperature, oocytes were incubated with different primary antibodies overnight at 4°C: Phospho-Ezrin (Thr567)/Radixin (Thr564)/Moesin (Thr558) (48G2) Rabbit mAb (CST, 3726, 1:300); Phospho-Myosin Light Chain 2 (Ser19) Mouse mAb (CST, 3675, 1:300); rabbit polyclonal anti-Mad2 (Biolegend, PRB-452C, 1:200); Purified Mouse anti-human BubR1 (BD Transduction Laboratories, 612502, 1:100); human anti-centromere (Immunovision, HCT-0100, 1:200), mouse monoclonal anti-alpha-tubulin with FITC (Sigma, F2168, 1:400). Oocytes were further incubated with FITC-conjugated Affinipure Goat Anti-Rabbit IgG (H + L) (proteintech, SA00003-2, 1:200), FITC-conjugated Affinipure Goat Anti-Mouse IgG (H + L) (proteintech, SA00003-1, 1:200) or anti-human-Cy3 (Jackson ImmunoResearch, AB-2340538, 1:200) for 1 h at room temperature. For F-actin staining, oocytes were incubated with Phalloidin-TRITC (Sigma, P1951, 5 μg/ml) at 4°C for overnight. Finally, oocytes were stained with 4′,6-diamidino-2-phenylindole (DAPI) for 5 min at room temperature. To conduct chromosome spread, the zona pellucida was removed by 0.5% pronase. Then oocytes were fixed in a medium of 1% paraformaldehyde in distilled H_2_O containing 0.15% Triton X-100 and 3 mM dithiothreitol. After air drying, the chromosome was stained with DAPI for 10 min. Fluorescent images were taken with laser scanning confocal microscopy (A1 Cell Imaging System; Nikon) on single planes under the same staining procedure and confocal microscopy parameters. Then, fluorescence intensity was assessed using NIS-Elements AR software (Nikon Instruments, Tokyo, Japan). Measurements of vitrified and vitrified + ConA were standardized with fresh group data. Specifically, the relative fluorescence intensity is the ratio of the experimental group fluorescence intensity to the average fluorescence intensity of the fresh group.

### Quantification of Mad2 and BubR1 Fluorescence Signal

The fluorescence intensity of Mad2 and BubR1 was quantified according to the previous study (Zhou et al., 2020): Fluorescence intensity was randomly measured and was quantified by drawing a circle of the dot-like CREST staining that includes SAC protein staining. The intensity of SAC proteins was normalized against the CREST fluorescence intensity.

### Quantitative Reverse Transcription PCR

Quantitative reverse transcription PCR (qRT-PCR) was performed by using an Applied Biosystems Step One Plus System and Power SYBR Green PCR Master Mix (TransGen Biotech). RNA was extracted from 50 oocytes using QIAGEN RNeasy Mini Kit, and cDNA was generated by using High Capacity cDNA Reverse Transcription Kit (Applied Biosystems). qRT-PCR was performed using an ABI 7500 real-time PCR instrument and a Fast 96-well Thermal Cycler (Applied Biosystems, Foster City, CA, United States). The sequences of all primers used are listed in Supplementary Table S1. GAPDH was used as a reference gene. The relative expression of genes was calculated with the comparative threshold cycle (CT) method as 2^−△△CT^.

### Statistical Analysis

All percentages or values from at least three repeated experiments were expressed as mean ± SEM. Data were analyzed by unpaired-samples *t*-test, provided by GraphPad Prism eight statistical software. The level of significance was accepted as *p* < 0.05.

## Results

### Vitrification Induced a Decline in Cortical Tension in Oocytes

**FIGURE 1:**
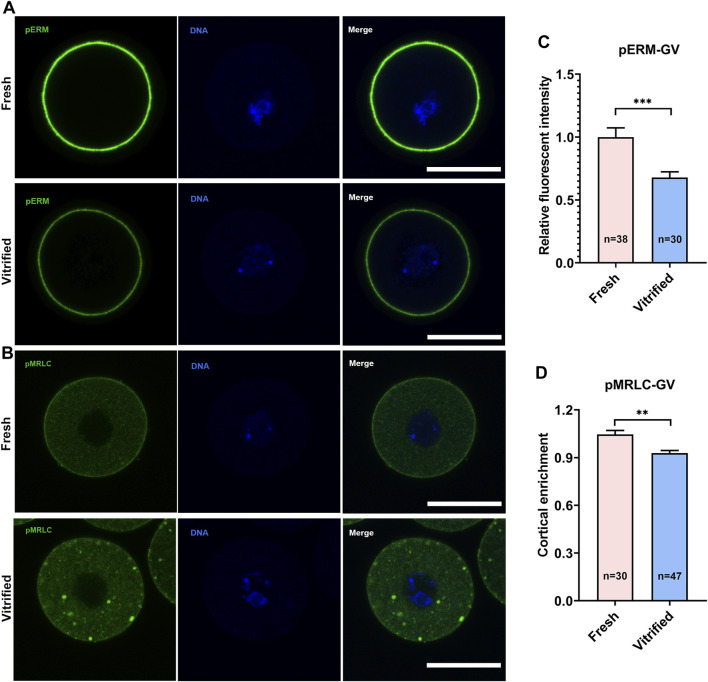
Vitrification induced a significant decline in cortical tension in GV oocytes. **(A)** Representative images of pERM staining in GV oocytes. Scale bar = 50 μm. **(B)** Representative images of pMRLC staining in GV oocytes. Scale bar = 50 μm. **(C)** Quantification of the relative pERM fluorescence intensity in fresh (*n* = 38) and vitrified (*n* = 30) GV oocytes. **(D)** Quantification of the ratio of cortical to cytoplasm pMRLC fluorescence intensity in fresh (*n* = 30) and vitrified (*n* = 47) GV oocytes. Data were presented as mean percentage (mean ± SEM) of at least three independent experiments. *p < 0.05, **p < 0.01, ***p < 0.001.

**FIGURE 2:**
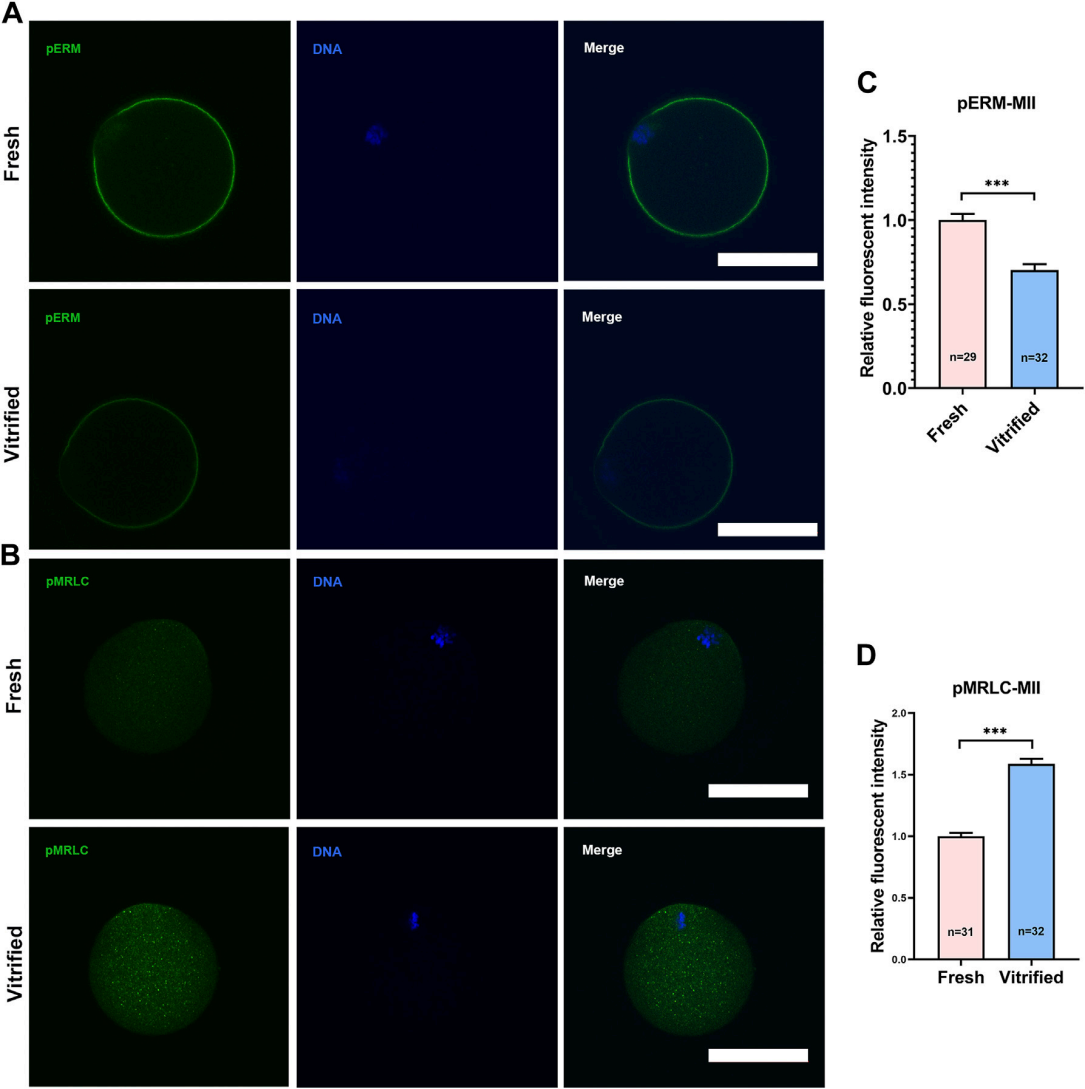
Vitrification induced a significant decline in cortical tension in MII oocytes. **(A)** Representative images of pERM staining in MII oocytes. Scale bar = 50 μm. **(B)** Representative images of pMRLC staining in MII oocytes. Scale bar = 50 μm. **(C)** Quantification of the relative pERM fluorescence intensity in fresh (*n* = 29) and vitrified (*n* = 32) MII oocytes. **(D)** Quantification of the relative cytoplasmic pMRLC fluorescence intensity in fresh (*n* = 31) and vitrified (*n* = 32) MII oocytes. Data were presented as mean percentage (mean ± SEM) of at least three independent experiments. *p < 0.05, **p < 0.01, ***p < 0.001.

To verify whether cortical tension was altered in vitrified oocytes, pERM and pMRLC were examined (Figures 1A,B, 2A,B). pERM was significantly decreased in GV vitrified oocytes (Fresh: 1 ± 0.07 *vs*. Vitified: 0.70 ± 0.03, *p* < 0.001; Figure 1C). pERM was also significantly decreased in MII vitrified oocytes (Fresh: 1 ± 0.04 *vs.* Vitified: 0.68 ± 0.04, *p* < 0.001; Figure 2C). Moreover, the cortical enrichment of pMRLC was significantly decreased in GV vitrified group (Fresh: 1.05 ± 0.03 *vs*. Vitified: 0.93 ± 0.02, *p* < 0.01; Figure 1D), and the distribution of pMRLC in cytoplasm was significantly increased in MII vitrified group (Fresh: 1 ± 0.03 *vs*. Vitified: 1.59 ± 0.04, *p* < 0.001; Figure 2D). These results indicated that cortical tension was decreased in vitrified oocytes.

### Cortical F-Actin Level was Not Affected After Vitrification

**FIGURE 3:**
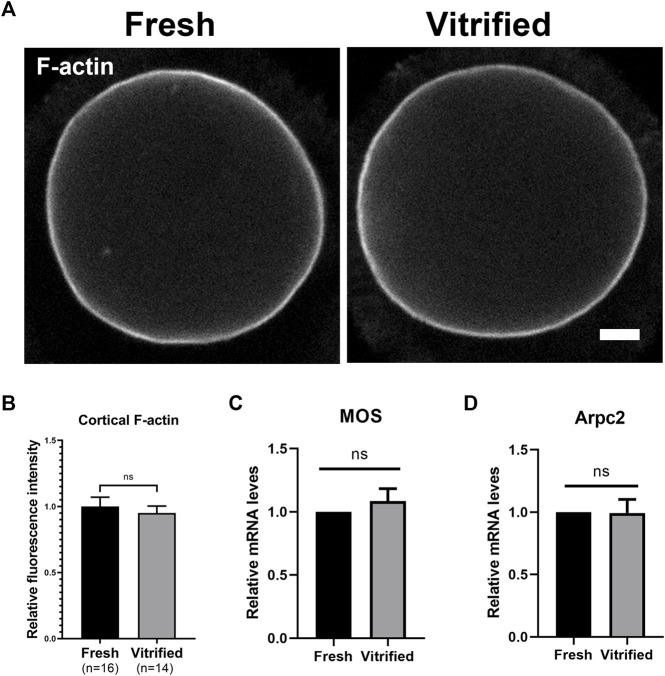
Cortical F-actin level was not affected after vitrification. **(A)** Representative images of the F-actin in fresh and vitrified oocytes after 9 h cultured. Scale bar = 10 μm. **(B)** The relative fluorescence intensity of cortical F-actin in fresh (*n* = 16) and vitrified (*n* = 14) groups. **(C)** The relative Mos mRNA level in BD + 3 h oocytes from different groups. **(D)** The relative Arpc2 mRNA level in BD + 3 h oocytes from different groups. Data were presented as mean percentage (mean ± SEM) of at least three independent experiments. *p < 0.05, **p < 0.01, ***p < 0.001, ns, no significant difference (*p* > 0.05).

In order to explore the level of F-actin, GV + 9 h oocytes were stained with Phalloidin-TRITC (Figure 3A). We found that cortical F-actin level was not affected after vitrification (Fresh: 1 ± 0.07 *vs*. Vitrified: 0.95 ± 0.05, *p* > 0.05; Figure 3B). In addition, the mRNA levels of Mos and Arpc2 were detected by qPCR, and the result showed that vitrification did not affect the expression of Mos (Fresh: 1 *vs*. Vitrified: 1.08 ± 0.10, *p* > 0.05; Figure 3C) and Arpc2 (Fresh: 1 *vs*. Vitrified: 0.90 ± 0.04, *p* > 0.05; Figure 3D).

### ConA Effectively Elevated Cortical Tension in Vitrified Oocytes

**FIGURE 4:**
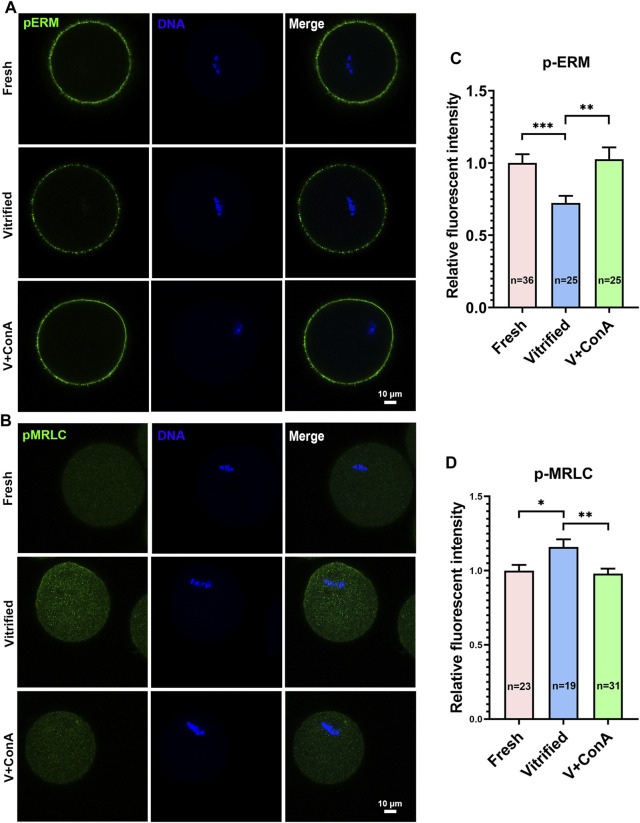
ConA effectively elevated cortical tension in vitrified oocytes. **(A)** Representative images of pERM staining in metaphase I (GV + 8 h) oocytes. Scale bar = 10 μm. **(B)** Representative images of pMRLC staining in metaphase I oocytes. Scale bar = 10 μm. **(C)** Quantification of the relative pERM fluorescence intensity in the fresh (*n* = 36), vitrified (*n* = 25) and V + ConA (*n* = 25) groups. **(D)** Quantification of the relative cytoplasmic pMRLC fluorescence intensity in the fresh (*n* = 23), vitrified (*n* = 19) and V + ConA (*n* = 31) groups. Data were presented as mean percentage (mean ± SEM) of at least three independent experiments. *p < 0.05, **p < 0.01, ***p < 0.001.

In order to increase the cortical tension of vitrified oocytes, GV vitrified oocytes were cultured in medium with 10 μg/ml ConA to metaphase I stage (GV + 8 h). We found that the pERM level in vitrified group cannot be recovered spontaneously during culture (Fresh: 1 ± 0.07 *vs*. Vitified: 0.70 ± 0.03, *p* < 0.001), while it could be rescued after ConA treatment (Vitified: 0.70 ± 0.03 *vs.* Vitified + ConA: 1.03 ± 0.08, *p* < 0.01; Figures 4A,C). Similarly, the cytoplasmic pMRLC level in vitrified group also cannot be resumed to the level of fresh group (Fresh: 1 ± 0.04 *vs*. Vitified: 1.16 ± 0.05, *p* < 0.05), while it could be reversed after ConA treatment (Vitified: 1.16 ± 0.05 *vs*. Vitified + ConA: 0.98 ± 0.03, *p* < 0.01; Figures 4B,D). These results indicated that ConA could increase the cortical tension of vitrified oocytes during *in vitro* culture.

### Increasing Cortical Tension Improved the Developmental Capability of Vitrified Oocyte

**FIGURE 5:**
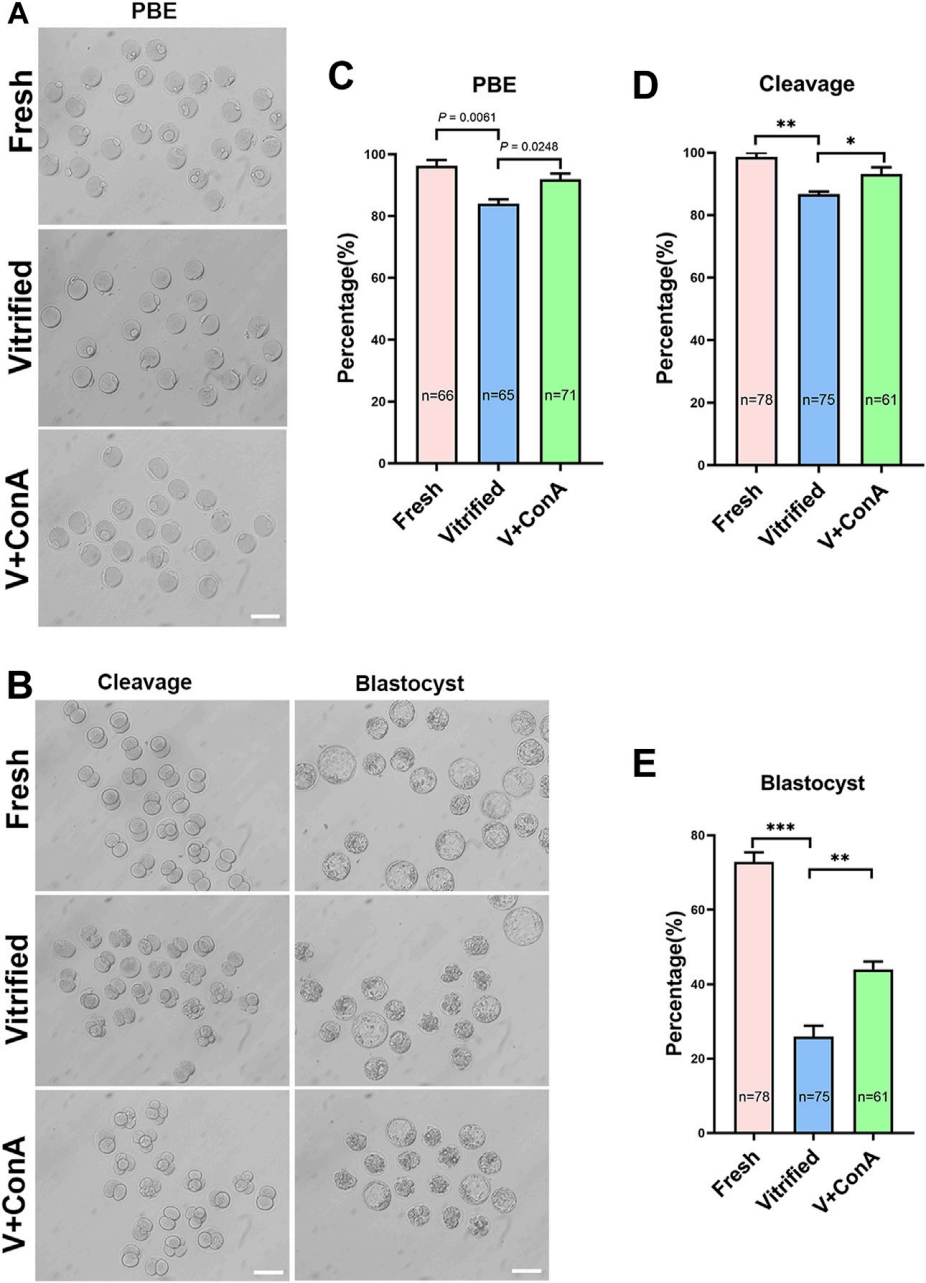
Increasing cortical tension improved the developmental capability of vitrified oocyte. **(A)** Representative images of polar body extrusion in the fresh, vitrified, and V + ConA groups. Scale bar = 50 μm. **(B)** Representative images of embryos development in the fresh, vitrified, and V + ConA groups. Scale bar = 50 μm. **(C)** The rate of polar body extrusion in the fresh (*n* = 66), vitrified (*n* = 65), and V + ConA (*n* = 71) groups. **(D)** The rate of cleavage in the fresh (*n* = 78), vitrified (*n* = 75), and V + ConA (*n* = 61) groups. **(E)** The rate of blastocyst in the fresh (*n* = 78), vitrified (n = 75), and V + ConA (*n* = 61) groups. Data were presented as mean percentage (mean ± SEM) of at least three independent experiments. *p < 0.05, **p < 0.01, ***p < 0.001.

To investigate whether increasing cortical tension of vitrified oocytes can improve oocyte and embryo development. GV vitrified oocytes were cultured in medium without or with ConA. We found that the polar body extrusion (PBE) rate of vitrified oocytes was significantly reduced (Fresh: 96.29 ± 1.87% *vs*. Vitrified: 83.99 ± 1.38%, *p* = 0.0061), while increasing the cortical tension could significantly increase the PBE rate (Vitified: 83.99 ± 1.38% *vs*. Vitified + ConA: 91.94 ± 1.80%, *p* = 0.0248; Figures 5A,C). Then, vitrified-thawed MII oocytes were cultured 1 h in medium without or with ConA before performing parthenogenetic activation. During the subsequent embryo development, both the cleavage rate (Fresh: 98.67 ± 1.33% *vs*. Vitrified: 86.72 ± 0.82%, *p* < 0.01) and blastocyst rate (Fresh: 72.86 ± 2.56% *vs*. Vitrified: 25.91 ± 2.96%, *p* < 0.001) were remarkable compromised in vitrified oocytes, while ConA treatment would significantly increase the cleavage rate (Vitrified: 86.72 ± 0.82% *vs*. Vitrified + ConA: 93.15 ± 2.15%, *p* < 0.05; Figures 5B,D) as well as the blastocyst formation rate (Vitrified: 25.91 ± 2.96% *vs*. Vitrified + ConA: 43.94 ± 2.14%, *p* < 0.01; Figures 5B,E).

### Increasing Cortical Tension Attenuated Aneuploidy Rate in Vitrified Oocytes

**FIGURE 6:**
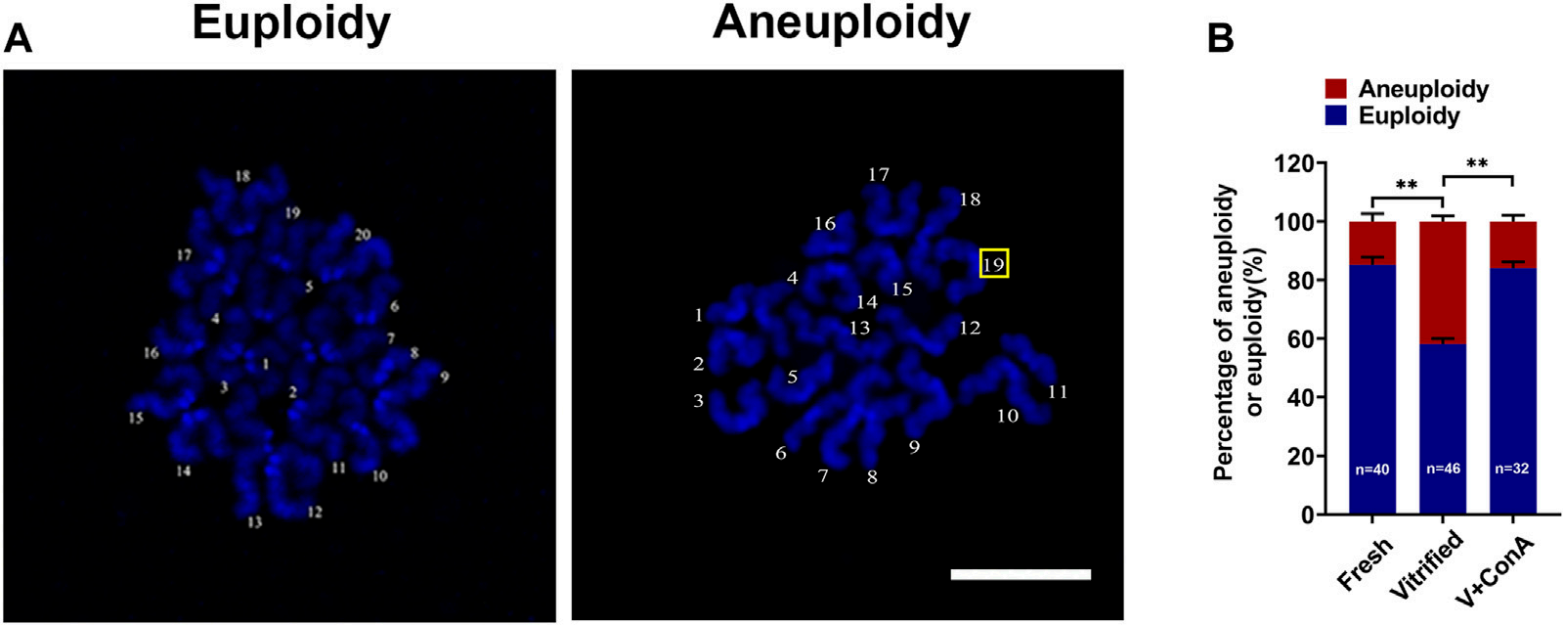
Increasing cortical tension attenuated aneuploidy rate in vitrified oocytes. **(A)** Representative images of euploidy and aneuploidy. Scale bar = 25 μm. **(B)** Rate of euploidy and aneuploidy oocytes in the fresh (*n* = 40), vitrified (*n* = 46) and V + ConA (*n* = 32) groups. Data were presented as mean percentage (mean ± SEM) of at least three independent experiments. *p < 0.05, **p < 0.01, ***p < 0.001.

To investigate whether increasing cortical tension of vitrified oocytes can decrease the aneuploidy rate, chromosome numbers in different groups was recorded by chromosome spread (Figure 6A). The aneuploidy rate was significant higher in vitrified oocytes (Fresh: 14.75 ± 2.63% *vs*. Vitrified: 41.82 ± 1.82%, *p* < 0.01), while increasing the cortical tension could correct it (Vitrified: 41.82 ± 1.82% *vs*. Vitrified + ConA: 15.87 ± 2.08%, *p* < 0.01; Figure 6B).

### Increasing Cortical Tension Promoted Spindle Positioning in Vitrified Oocytes

**FIGURE 7:**
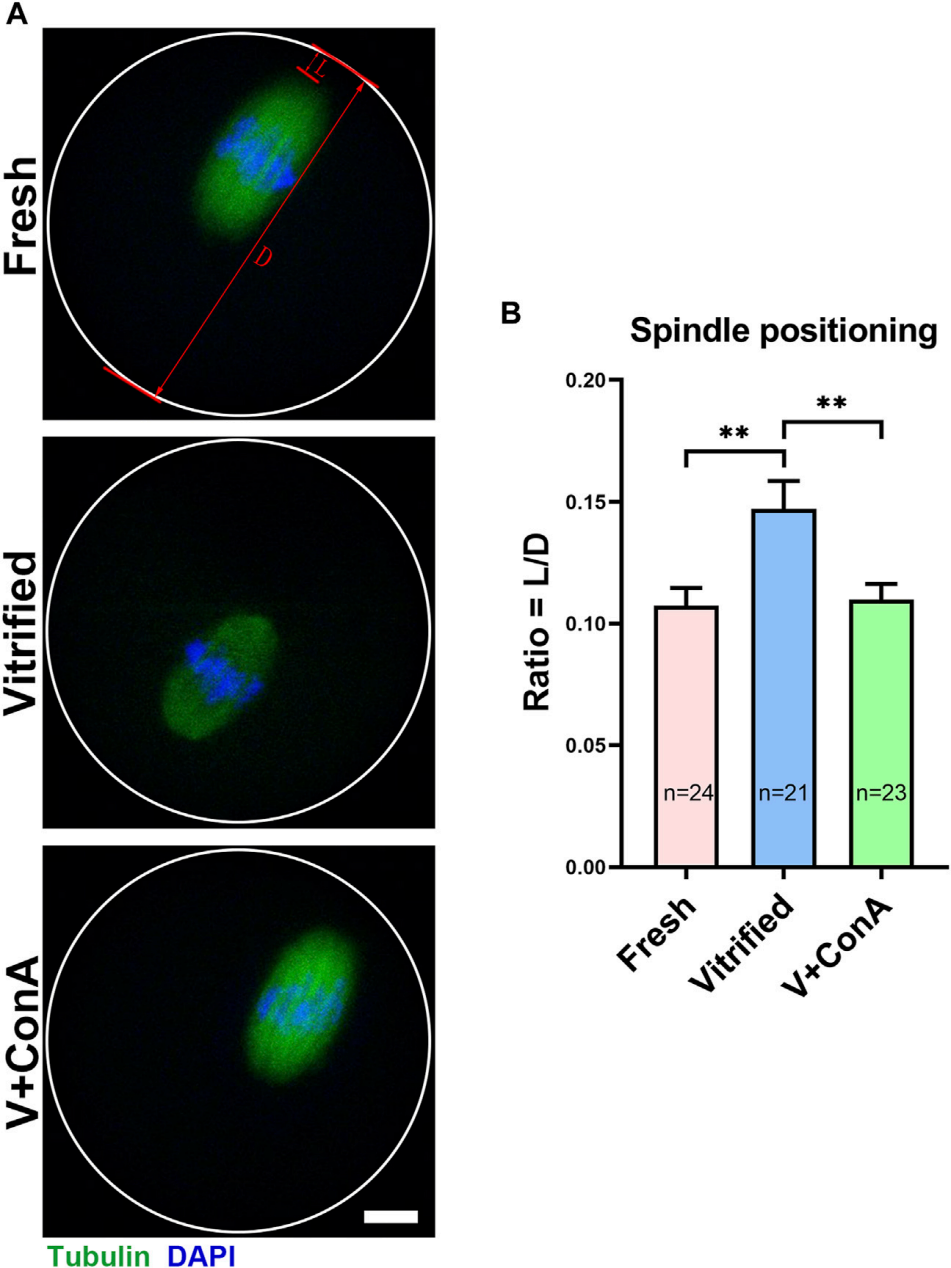
Increasing cortical tension promoted spindle positioning in vitrified oocytes. **(A)** Representative images of the spindle positioning in the fresh, vitrified, and V + ConA oocytes after 9 h culture. Scale bar = 10 μm. **(B)** Quantitative analysis of the spindle positioning in the fresh (*n* = 24), vitrified (*n* = 21), and V + ConA (*n* = 23) groups. L, length between spindle leading pole and cortex; D, diameter of oocyte. Spindle positioning was defined by the raito of length between spindle leading pole and cortex to diameter of oocyte. Data were presented as mean percentage (mean ± SEM) of at least three independent experiments. *p < 0.05, **p < 0.01, ***p < 0.001.

In order to investigate whether the spindle positioning was affected in vitrified oocyte, spindle positioning was detected at GV + 9 h (Figure 7A). Then, spindle positioning was quantified according to the previous study (Pan et al., 2021). We found that the relative distance between the spindle leading pole and cortex of vitrified oocytes was significantly increased (Fresh: 0.11 ± 0.01 *vs*. Vitrified: 0.15 ± 0.01, *p* < 0.01), while increasing cortical tension could significantly decrease the distance (Vitrified: 0.15 ± 0.01 *vs*. Vitified + ConA: 0.11 ± 0.01, *p* < 0.01; Figure 7B). These results suggest that increasing cortical tension of vitrified oocytes could promote spindle positioning.

### Increasing Cortical Tension Regulated Microtubule Dynamics During Chromosome Segregation

**FIGURE 8:**
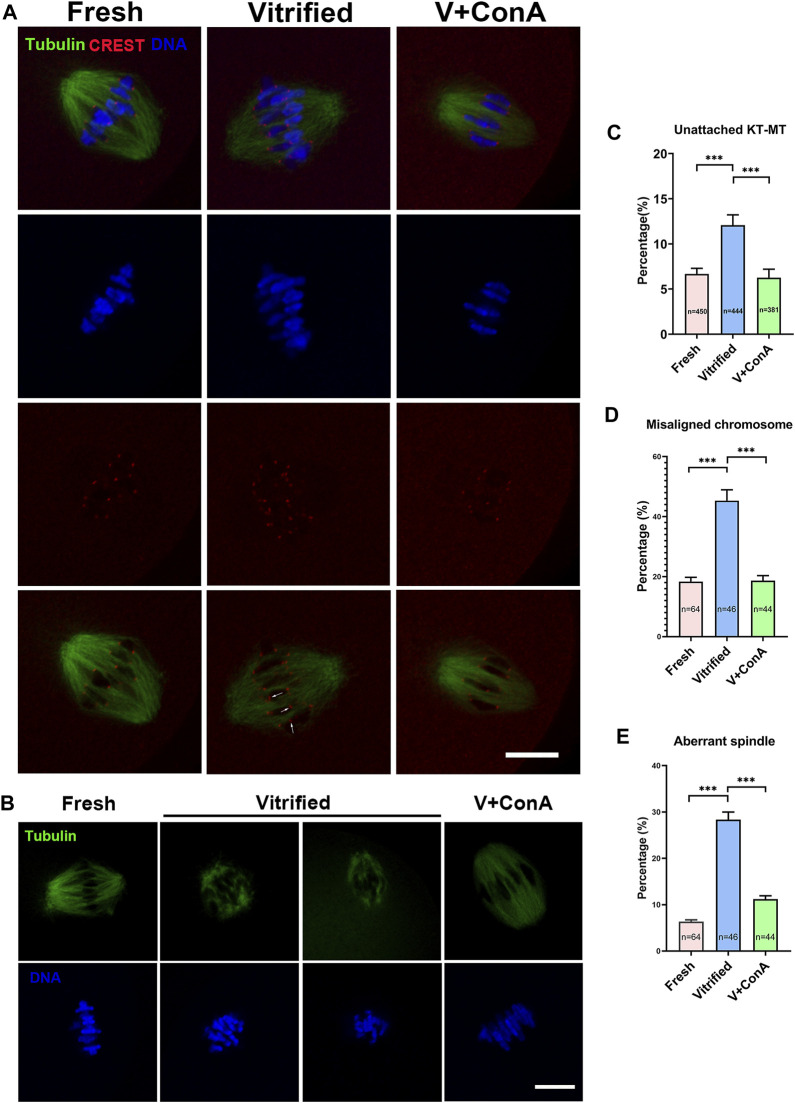
Increasing cortical tension regulated microtubule dynamics during chromosome segregation. **(A)** KT-MT attachment in different groups at 6 h after GVBD. White arrows indicate nonconnected kinetochores. Scale bar = 25 μm. **(B)** Representative images of spindle morphologies and chromosome alignment in fresh, vitrified, and V + ConA oocytes at 6 h after GVBD. Scale bar = 25 μm. **(C)** The rate of unattached KT-MT in the fresh (*n* = 450), vitrified (*n* = 444), and V + ConA (*n* = 381) groups. **(D)** The rate of misaligned chromosome in the fresh (*n* = 64), vitrified (*n* = 44), and V + ConA (*n* = 46) groups. **(E)** The rate of aberrant spindle in the fresh (*n* = 64), vitrified (*n* = 44), and V + ConA (*n* = 46) groups. Data were presented as mean percentage (mean ± SEM) of at least three independent experiments. *p < 0.05, **p < 0.01, ***p < 0.001.

In order to investigate whether increasing cortical tension of vitrified oocytes can affect microtubule dynamics, kinetochores, microtubules and chromosomes were stained at BD (breakdown) + 6 h (Figure 8A). We found that vitrification induced significant higher proportion of unattached KT-MT (Fresh: 6.68 ± 0.62% *vs*. Vitrified: 12.08 ± 1.15%, *p* < 0.001), whereas increasing cortical tension could rescue this phenomenon (Vitrified: 12.08 ± 1.15% *vs*. Vitified + ConA: 6.26 ± 0.93%, *p* < 0.001; Figure 8C). Moreover, the rate of misaligned chromosome (Fresh: 18.37 ± 1.41% *vs*. Vitified: 45.35 ± 3.61%, *p* < 0.001) and aberrant spindle proportion (Fresh: 6.33 ± 0.40% *vs*. Vitrified: 28.33 ± 1.67%, *p* < 0.001) were dramatically elevated in vitrified oocytes. Notably, ConA treatment could alleviate chromosome misalignment (Vitrified: 45.35 ± 3.61% *vs*. Vitrified + ConA: 18.67 ± 1.68%, *p* < 0.001; Figures 8B,D), and also reduce aberrant spindle rate (Vitrified: 28.33 ± 1.67% *vs*. Vitrified + ConA: 11.20 ± 0.72%, *p* < 0.001; Figures 8B,E) in vitrified oocytes. These results showed that increasing cortical tension could improve the KT-MT attachment, chromosome alignment and spindle morphology of vitrified oocytes.

### Increasing Cortical Tension Strengthened SAC Activity

**FIGURE 9:**
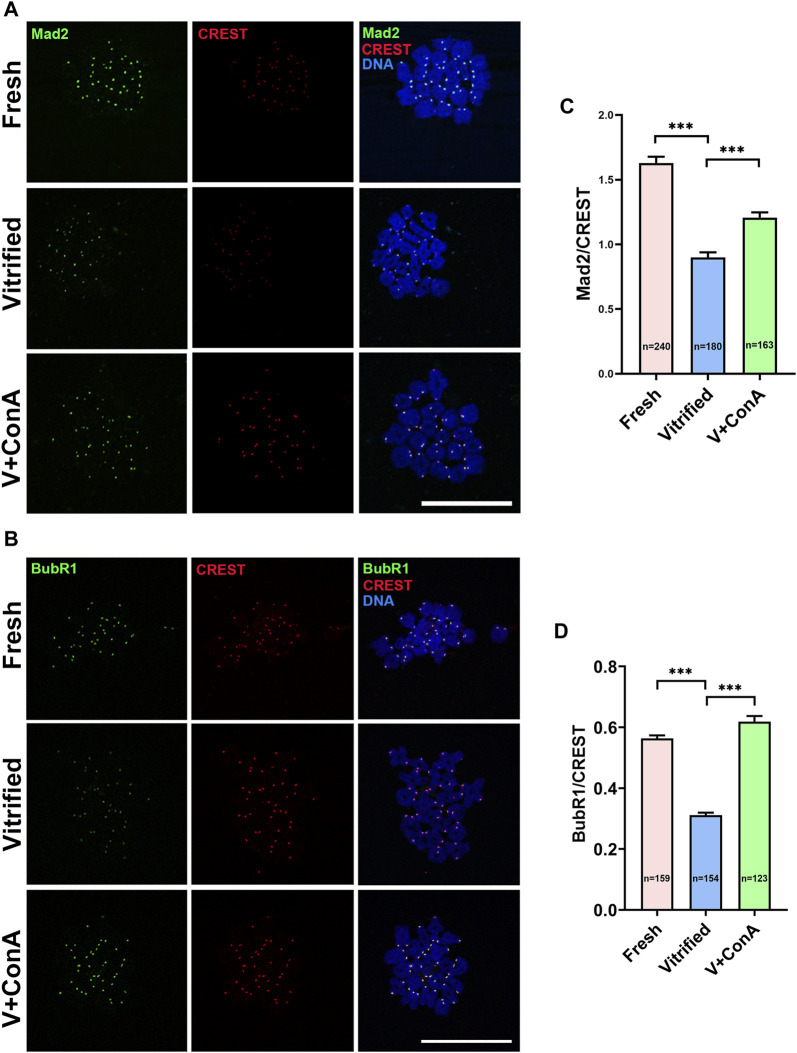
Increasing cortical tension strengthened SAC activity. **(A)** The localization of Mad2 at prometaphase I stage in fresh, vitrified, and V + ConA oocytes. At 3 h after germinal vesicle breakdown (GVBD), oocytes were immunostained for Mad2, CREST, and DNA (DAPI). Scale bar = 50 μm. **(B)** The localization of BubR1 at prometaphase I stage in the fresh, vitrified, and V + 10 μg/ml ConA oocytes. At 3 h after GVBD, oocytes were stained for BubR1, CREST, and DNA (DAPI). Scale bar = 50 μm. **(C)** The relative fluorescence intensity of Mad2 to CREST was measured in fresh (*n* = 240), vitrified (*n* = 180), and V + ConA (*n* = 163) groups. The signal intensity of Mad2 was normalized with that of CREST. **(D)** The relative fluorescence intensity of BubR1 to CREST was measured in the fresh (*n* = 159), vitrified (*n* = 154), and V + ConA (*n* = 123) groups. The signal intensity of BubR1 was normalized with that of CREST. Data were presented as mean percentage (mean ± SEM) of at least three independent experiments. *p < 0.05, **p < 0.01, ***p < 0.001.

Then, two essential SAC proteins [mitotic arrest deficient 2 (Mad2) and Bub1-related protein 1 (BubR1)] were stained and intensities were measured with CREST as a reference indicator (Figures 9A,B). Compared with the fresh group, the signal intensities of Mad2 (Fresh: 1.63 ± 0.05 *vs*. Vitrified: 0.90 ± 0.04, *p* < 0.001) and BubR1 (Fresh: 0.56 ± 0.01 *vs*. Vitrified: 0.31 ± 0.01, *p* < 0.001) were remarkably decreased after vitrification. When treated with ConA, the fluorescence intensities of Mad2 (Vitrified: 0.90 ± 0.04 *vs*. Vitrified + ConA: 1.21 ± 0.04, *p* < 0.001; Figure 9C) and BubR1(Vitrified: 0.31 ± 0.01 *vs*. Vitrified + ConA: 0.62 ± 0.02, *p* < 0.001; Figure 9D) were significantly increased in vitrified oocytes. The results indicated that increasing the cortical tension could enhance SAC activity.

## Discussion

Oocyte underwent a dramatic osmotic pressure change during the vitrification/thawing process, which resulted in a drastic change in the morphology of the oocyte (Gallardo et al., 2019; Wang et al., 2020). To investigate whether this severe deformation could lead to changes in oocyte cortical tension, we examined the expression of two proteins, pERM and pMRLC, which play an important role in oocyte cortical tension regulation. It has been confirmed that the level of pERM protein in oocytes is consistent with the change of cortical tension (Larson et al., 2010). In this study, pERM protein levels of vitrified GV and MII oocytes were decreased (Figures 1C, 2C), which indicated that cortical tension of frozen oocytes were decreased. The distribution of pMRLC during meiosis is more complicated than pERM. pERM is consistently distributed in the cortex during meiosis, whereas pMRLC is gradually expelled from the cortex into the cytoplasm. Therefore, the cortical tension change was reflected by cortical enrichment of pMRLC in GV oocytes, while in regard to MII stage oocytes, it was reflected by cytoplasmic pMRLC. Specifically, cortical pMRLC has a positive correlation with cortical tension while cytoplasmic pMRLC has a negative correlation with cortical tension (Chaigne et al., 2013; Bennabi et al., 2020). The cortical enrichment of pMRLC in vitrified GV oocytes was decreased (Figure 1D), and the cytoplasmic pMRLC in vitrified MII oocytes was increased (Figure 2D). These change patterns of pMRLC are consistent with the decreased cortical tension model. Thus, vitrification could decrease the cortical tension of oocytes.

Previous research found that cortical tension decreases during oocytes meiosis progression (Larson et al., 2010). The mechanism lies in that the up-regualted of Mos would activate Arp2/3 at BD + 3 h, and eventually result in cortical F-actin increase and spatially discharging Myosin II from cortex into cytoplasm (Nakanishi et al., 2007; Mendoza et al., 2011). To investigate whether the F-actin levels in vitrified oocytes were changed. We detected the level of F-actin at GV + 9 h, we found that the cortical F-actin density was not significantly different between vitrified and fresh groups (Figure 3B). Then, we examined the expression of Mos and Arpc2 at BD + 3 h, and the result showed that vitrification did not affect the mRNA levels of Mos and Arpc2 (Figures 3C,D). Furthermore, these results indicated that the cortical F-actin was not affected after vitrification.

Concanavalin A (ConA) is a tetravalent lectin that crosslinks the cell surface through binding to membrane glycosylated proteins (Chaigne et al., 2013). Several studies have used ConA to increase oocyte cortical tension (Larson et al., 2010; Chaigne et al., 2013), suggesting that the effect of ConA is stable. 100 μg/ml ConA treatment caused a 69% increase in cortical tension of fresh MII oocytes (Lee et al., 2009). However, excessive cortical tension in fresh oocytes resulted in impaired spindle migration and increased aneuploidy rate (Chaigne et al., 2013). Therefore, we added 10 μg/ml ConA during *in vitro* culture of vitrified oocytes. A study reported that the localizations of pMRLC and phosphorylated ERM were not dramatically different between controls and ConA-treated eggs (Larson et al., 2010). But this study did not measure the fluorescence intensity of pERM and pMRLC. In our study, ConA did not change the localizations of these two proteins also, and their changes in fluorescence intensity could indicate that 10 μg/ml ConA increased cortical tension (Figure 4).

Then, we investigated the effect of cortical tension on oocyte development. Our results indicated that the polar body extrusion (PBE) rate of vitrified oocytes was decreased, while increasing the cortical tension could increase the PBE rate (Figure 5C). Fresh oocytes treated with 100 μg/ml ConA showed a decreased PBE rate, which could be rescued by succinyl-concanavalin A, an antagonist of ConA (Chaigne et al., 2013). Combined with our study, it can be speculated that there is a narrow window of cortical tension for normal polar body extrusion, too low or too high cortical tension will lead to disruptions in PBE. In addition, increasing the cortical tension could improve the subsequent development of vitrified oocytes (Figures 5D,E). The mechanical properties of zygote have an important impact on the developmental potential of human embryos (Yanez et al., 2016). What’s more, cell surface forces can influence the differentiation of embryonic stem cells (Bergert et al., 2021; De Belly et al., 2021; Muncie and Weaver, 2021). We speculate that increasing cortical tension of vitrified oocytes could affect mechanical property of zygotes and cell fate determination, thus improved embryo development. Interestingly, the cortical tension of post-ovulatory aging oocytes was also decreased (Mackenzie et al., 2016). Whether increasing the cortical tension of post-ovulatory aging oocytes could improve oocytes and subsequent embryos development deserves a further study.

Our previous study showed that aneuploidy rate was significantly increased in vitrified oocytes (Cheng et al., 2014). In this study, we found that increasing the cortical tension of vitrified oocytes could decrease the aneuploidy rate (Figure 6B). It was reported that in mouse oocytes, reduction of cortical tension by the expression of cVAC (cortical verprolin-homology coflilin-homology acidic) or cFH1FH2 (cortical FH1FH2 nucleating domain of formin 2) would induce abnormal high aneuploidy rate (Bennabi et al., 2020). Our results further confirmed that cortical tension was involved in maintaining oocyte euploidy. In addition, we discovered that the distance between spindle leading pole and cortex was increased in vitrified oocyte, which could be shortened by increasing the cortical tension (Figure 7B). This indicated that the decreased cortical tension was contributed to the impaired spindle positioning in vitrified oocytes, and our data was consistent with the previous finding that proper cortical tension was essential for normal spindle positioning (Chaigne et al., 2015).

In our study, unconnected KT-MT was increased in vitrified oocytes, while increasing the cortical tension could rescue this phenomenon (Figure 8). The result implied that proper KT-MT was essential for normal meiosis progression, which was consistent with the previous finding that accurate chromosome separation depends on the correct attachment between microtubule and kinetochore (Nakagawa and FitzHarris, 2017). The decrease of SAC activity weakened checkpoint monitoring, resulting in unattached KT-MT or other attachment errors that cannot be corrected (Skoufias et al., 2001). Then we detected the levels of Mad2 and BubR1, which are two important components of SAC (Homer, 2006; Musacchio, 2015). Mad2 and BubR1 were both decreased in vitrified oocytes, indicating that the SAC activity was decreased. Nevertheless, increasing the cortical tension could strengthen the SAC activity in vitrified oocytes (Figure 9). A study reported that kinetochore stretching promoted SAC silencing by PP1 recruitment stimulation (Uchida et al., 2021). Whether cortical tension can affect SAC activity through kinetochore stretching needs to be further studied. To sum up, increasing cortical tension of vitrified oocytes could enhance SAC activity, and the activated checkpoint monitoring could facilitate proper KT-MT attachment, which in turn ensures correct chromosomes separation and maintains euploidy in oocytes. This is the first study revealed that vitrification decreased the oocyte cortical tension. Furthermore, increasing the cortical tension of mouse vitrified oocyte could decrease aneuploidy rate through improving SAC activity and KT-MT attachment.

## Conclusion

In conclusion, vitrification decreased the cortical tension of mouse oocytes. Artificially increasing cortical tension in vitrified oocyte could increase oocyte and embryo development. Furthermore, it could improve spindle positioning, strengthen SAC activity and ensure KT-MT attachment. Importantly, cortical tension could regulate aneuploidy rate through influencing SAC activity and KT-MT attachment. Our results will provide an interdisciplinary perspective into understanding the cryoinjuries in mouse oocytes, and provide a reasonable theoretical basis for delineating the mechanism underlying cortical tension-regulated meiotic progression.

## Data Availability

The original contributions presented in the study are included in the article/Supplementary Material, further inquiries can be directed to the corresponding author.
