# Supplementary material for: Artificially Increasing Cortical Tension Improves Mouse Oocytes Development by Attenuating Meiotic Defects During Vitrification
Source: Front Cell Dev Biol. 2022 Mar 24;10:876259. doi: 10.3389/fcell.2022.876259 (PMC8987233; doi:10.3389/fcell.2022.876259)
Supplement: Supplementary file 2 [file Table1.DOCX]

**Supplemental Material**

**Table S1.** Primer sequences are used for quantitative real-time PCR.

| Gene | Primer sequence (5ʹ–3ʹ) | Product size (bp) | NCBI Sequences |
| --- | --- | --- | --- |
| *GAPDH* | F: TGGCCTTCCGTGTTCCTAC  R: GAGTTGCTGTTGAAGTCGCA | 178 | NM_001289726.1 |
| *Mos* | F: ATAAAGCCACTTACCACGGTG  R: CAATGTTCAGTTCAGCCCAGAA | 105 | NM_020021.3 |
| *Arpc2* | F: CCTGGAAAATCTACCTGCATCC  R: AAACCGTGCTGAAGACTACTG | 212 | NM_001357387.1 |

Abbreviation: PCR, polymerase chain reaction; *GAPDH*, glyceraldehyde-3-phosphate dehydrogenase; *Mos*, Moloney sarcoma oncogene; *Arpc2*, Actin related protein 2/3 complex, subunit 2.
